# Absence of Excess Intra-Individual Variability in Retinal Function in People With Schizophrenia

**DOI:** 10.3389/fpsyt.2020.543963

**Published:** 2020-11-20

**Authors:** Samantha I. Fradkin, Molly A. Erickson, Docia L. Demmin, Steven M. Silverstein

**Affiliations:** ^1^Department of Psychology, Rutgers University, Piscataway, NJ, United States; ^2^University Behavioral Health Care, Rutgers University, Piscataway, NJ, United States; ^3^Department of Psychology, University of Rochester, Rochester, NY, United States; ^4^Department of Psychiatry, University of Chicago, Chicago, IL, United States; ^5^Departments of Psychiatry and Ophthalmology, Rutgers University, Piscataway, NJ, United States; ^6^Departments of Psychiatry, Neuroscience, and Ophthalmology, and Center for Visual Science, University of Rochester Medical Center, Rochester, NY, United States

**Keywords:** intra-individual variability, retina, electroretinography (ERG), schizophrenia, sensory processing

## Abstract

People with schizophrenia exhibit increased intra-individual variability in both behavioral and neural signatures of cognition. Examination of intra-individual variability may uncover a unique functionally relevant aspect of impairment that is not captured by typical between-group comparisons of mean or median values. We and others have observed that retinal activity measured using electroretinography (ERG) is significantly reduced in people with schizophrenia; however, it is currently unclear whether greater intra-individual variability in the retinal response can also be observed. To investigate this, we examined intra-individual variability from 25 individuals with schizophrenia and 24 healthy controls under two fERG conditions: (1) a light-adapted condition in which schizophrenia patients demonstrated reduced amplitudes; and (2) a dark-adapted condition in which the groups did not differ in amplitudes. Intraclass correlation coefficients (ICC) were generated to measure intra-individual variability for each subject, reflecting the consistency of activation values (in μv) across all sampling points (at a 2 kHz sampling rate) within all trials within a condition. Contrary to our predictions, results indicated that the schizophrenia and healthy control groups did not differ in intra-individual variability in fERG responses in either the light- or dark-adapted conditions. This finding remained consistent when variability was calculated as the standard deviation (SD) and coefficient of variation (CV) of maximum positive and negative microvolt values within the a- and b-wave time windows. This suggests that although elevated variability in schizophrenia may be observed at perceptual and cognitive levels of processing, it is not present in the earliest stages of sensory processing in vision.

## Introduction

People with schizophrenia often demonstrate increased variability in performance on cognitive tasks relative to control groups, and in stimulus-driven electrophysiological activation patterns [e.g., (1, 2)]. Although the underlying neural mechanisms remain unclear, computational models suggest that response variability may reflect weak signal-to-noise ratio in the brain and excessive random neural firing (3). Therefore, rather than view excessive variability as simply error variance, continued study of this phenomenon can provide insights into mechanisms of the disorder that are typically obscured in studies solely focused on between-group differences in mean or median values.

Intra-individual variability is typically operationalized as variability within an individual's responses to the same stimulus during a single task session, or to the same task across multiple sessions. In people with schizophrenia, excessive within-task intra-individual variability has been demonstrated behaviorally in reaction time and time-estimation tasks (1, 4, 5). In particular, Rentrop et al. (6) observed increased intra-individual variability in a high-functioning sample of people with schizophrenia, within the context of normal accuracy and mean reaction time values in behavioral tasks, which underscores how the investigation of intra-individual variability can elucidate information concealed in between-group analyses of measures of central tendency. Increased intra-individual variability has also been demonstrated in electrophysiological indices such as the mismatch negativity (MMN) (7), P3 amplitude and latency (8), and N2 latency (2). This includes increased intra-individual variability in the MMN within an ultra-high risk sample (9), suggesting that it may be a core feature of schizophrenia-spectrum disorders and not an artifact of treatment or chronicity-related factors.

The purpose of this study was to determine if abnormally increased intra-individual variability is observable in retinal responses in people with schizophrenia. Data on retinal structure and function are often considered to be proxies for indices of brain structure and function given that the retina and brain share similar origins during embryonic development, as well as similar cell types (e.g., neurons, glial cells), neurotransmitters, a layered architecture, and bidirectional synaptic connections, but the retina is a more accessible component of the central nervous system than the brain (10). Flash electroretinography (fERG) studies have shown that, compared to healthy controls, people with schizophrenia display amplitude reductions in the a- and b-wave, which reflect weak photoreceptor and bipolar-Müller cell function, respectively, in both photopic (light-adapted) and scotopic (dark-adapted) conditions [see (11–13) for reviews]. To date, however, all fERG studies in schizophrenia have focused on mean between-group differences in ERG indices, and so the degree of intra-individual variability in retinal responses, and how this relates to amplitude reductions, is unknown. Given that retinal cells provide the earliest input to the visual system (14), the importance of studying stability in the retinal response is two-fold: (1) ascertaining intra-individual variability in the ERG response can help determine the extent to which reduced ERG waveforms observed in schizophrenia reflect impoverished retinal cell function, and/or are a consequence of increased intra-individual variability; and (2) retinal responses occur earlier than processes studied previously, and so determining whether intra-individual variability is increased at the level of the first and second synapses can clarify the generalizability of intra-individual variability to sensory-level processing in schizophrenia and inform our understanding of downstream anomalies. For example, instability in the retinal response could lead to weaker and less predictable signals reaching the cortex, which may reduce precision and increase uncertainty (entropy) in early visual cortex activity, thereby contributing to compensatory and other sequelae such as visual distortions, increases in stimulus (but also noise) salience, misperceptions, inappropriate assessment of meaning and significance, and delusional interpretation of events (15–17).

To derive a measure of intra-individual variability in the retinal response, we computed the intraclass correlation (ICC), standard deviation (SD), and coefficient of variation (CV) from trial x trial data for two fERG conditions in a previously published study (18): (1) a photopic condition, in which we observed reductions in ERG waveform indices within the schizophrenia group; and (2) a weak light stimulus scotopic condition, where we did not find between-group differences in retinal activity. We hypothesized that the schizophrenia group would demonstrate greater intra-individual variability in both conditions compared to the healthy control group. We also assessed the relationships between variability in the retinal response, ERG parameters, and symptom severity scores.

## Materials and Methods

### Participants

As described in the original study (18), we recruited 25 individuals with schizophrenia from Rutgers University Behavioral Health Care programs as well as 24 healthy control participants, who were recruited through community advertisements. Demographic information for all participants can be found in Table 1. Exclusion criteria included: (1) outside the age range of 18–60; (2) presence of an active substance use disorder in the past 6 months; (3) history of diseases known to affect vision (e.g., diabetes, hypertension); (4) history of a visual condition or disease (e.g., strabismus, nystagmus, glaucoma), or an eye injury; (5) history of serious head injury with loss of consciousness >10 min; or (6) history of neurological disease. The protocol was approved by the Rutgers University Institutional Review Board and all subjects gave written informed consent.

**Table 1:** Individual demographic characteristics.

**Variable**	**SZ**	**CON**
*N*	25	24
**Gender**
Female	4	6
Male	21	18
Age (*M* ±*SD*)	36.80 ± 10.83	32.13 ± 11.92
**Race**
Caucasian	13	13
African American	7	7
Asian	5	3
Other	0	1
**Ethnicity**		
Hispanic	5	4
Non-Hispanic	20	20
Education (*M* ±*SD*)	13.32 ± 2.12	15 ± 1.98
**PANSS (*****M*** **±*****SD*****)**	
Positive	10.64 ± 4.09	
Negative	14.24 ± 5.21	
Disorganized	5.32 ± 2.27	
Excitement	6.92 ± 2.50	
Depression	13.08 ± 4.88	

*SZ, schizophrenia; CON, control; PANSS, Positive and Negative Syndrome Scale*.

### Clinical Assessments

Diagnostic criteria for patients were confirmed with the Structured Clinical Interview for *DSM-IV* Diagnosis (SCID), patient version (19), and the absence of psychotic symptoms and a current major depressive episode in healthy controls was confirmed through the SCID-IV non-patient edition (20). In order to assess symptom severity over the last 2 weeks, research staff also administered the Positive and Negative Syndrome Scale [PANSS (21)] to patients. A five-factor model was used to calculate symptom severity scores for positive, negative, disorganized, excitement, and depression factors (22, 23).

### fERG Protocol

We collected fERG using the RETeval, which is a portable FDA-approved hand-held device that does not require corneal contact or pupil dilation. Prior to testing, an alcohol swab was used to clean the skin of the lower eyelid. A sensor strip containing positive, negative, and ground electrodes was then placed 2 mm below the eye. The dome of the RETeval was placed over the participant's eye and the light flashes were delivered via the device. Although testing was conducted for right and left eyes, the analyses presented here focus on right eye data. To ensure constant retinal illumination, the RETeval uses Troland-based stimulation (Td), which accounts for changes in pupil size (which is measured continuously by the device), and is calculated as the product of photopic flash luminance [cd (s/m^2^)] and pupillary area (mm^2^) (24, 25).

The ERG tests included in these analyses were selected from the parent study, which included seven ERG tests completed consecutively (18). As part of the original protocol, participants first underwent a 5 min light-adaptation period in order to adjust their eyes to the room lighting. They then completed the first photopic ERG test, in which they viewed a series of 30 light flashes at a light intensity of 100 Td-s and frequency of 1 Hz. This is the first of the two conditions analyzed in the present study. The second ERG test for which we analyzed intra-individual variability was the first scotopic (dark-adapted) and fifth consecutive test of the original sequence. Participants completed a 10 min dark-adaptation prior to scotopic testing. This test included five light flash trials with a weak luminance of 2.8 Td-s and a frequency of 0.25 Hz. Flash ERG was recorded through the RETeval device for all of the tests described and data were collected at a sampling rate of ~2 kHz.

### fERG Analysis

Offline, data were preprocessed using MATLAB (26). The raw fERG was first filtered using EEGLAB (27) with a 1–100 Hz bandpass and 60 Hz notch filter. The data were then segmented into 120 ms epochs that included a 20 ms baseline period preceding each stimulus. An artifact rejection routine was then used to reject epochs with (a) differences between maximum and minimum values that exceeded 1 millivolt, or (b) microvolt values exceeding three SDs from the subject's mean for a given time point across all trials in the condition. We then visually inspected the data to remove any trials containing artifacts that were not detected using the algorithms described above. Finally, two schizophrenia patient subjects did not contribute data to the scotopic conditions. In particular, one subject did not engage in the scotopic condition after completing the photopic condition, and data from one subject was removed following artifact rejection procedures. For all data included in the final analyses, subjects with schizophrenia retained 94.27 ± 3.27% and 94.78 ± 0.09% of trials in the photopic and scotopic conditions, respectively, and healthy controls retained 92.36 ± 4.11% and 97.50 ± 6.89% of trials. The difference in number of trials retained between the two groups was not significant (*p*s > 0.08) for both conditions.

ERG parameters were measured as the amplitude of the a- and b-wave in microvolts (μv). The a-wave metric is calculated as the difference between the mean of pre-trial baseline activity and the first trough of the waveform, while the amplitude of the b-wave is measured as the difference between the positive peak of the b-wave and the trough of the a-wave (28). Implicit times (peak latency measurements) for each waveform are measured in milliseconds (ms). The a-wave implicit time parameter is measured as the time elapsed from the flash to the a-wave trough, while b-wave implicit time is calculated as the time elapsed from the flash to the b-wave peak (29).

### Intraindividual Variability

Intra-individual variability was operationalized as the intraclass correlation coefficient (ICC; two-way random model) for each condition for each subject. ICC here thus measures intra-individual variability in ERG responses across multiple identical light flashes, or, the degree to which the voltage changes across the duration of each trial relative to the variability in voltage across each time point, across all trials in the condition. Thus, the ICC equals the variance due to differences across trial duration divided by the sum of the variance due to differences in voltage at a given timepoint across all trials in a condition and variance due to error. Within this study, the ICC incorporated all timepoints following flash onset and did not include baseline activity. The ICC was generated using the Real Statistics Resource Pack software (Release 6.8) [Copyright (2013–2020) Charles Zaiontz. www.real-statistics.com]. It is equivalent to:

(1) $$\begin{array}{r} \frac{var\left(\beta \right)}{var\left(\alpha \right)+var\left(\beta \right)+var\left(\varepsilon \right)} \end{array}$$

Assuming that the data are represented such that all successive microvolt values within a single trial are listed in successive rows within a single column, and data from consecutive trials are represented in consecutive columns, then *var*(β) is equivalent to the variance due to differences in the microvolt values within single trials, or *(MS*_*Row*_ –* MS*_*E*_*)*/*k*. This is divided by all sources of variance, including *var*(α), which is equivalent to (*MS*_*Col*_ –* MS*_*E*_*/n)*, and variance due to error, or *var*(ε), which is equivalent to *MS*_*E*_ (30). The data is typically represented within a data matrix where the variables include *n*, which reflects the number of rows, or the number of samples of microvolt values across a single trial, and *k*, which represents the number of columns, or number of trials. The *MS* variable represents the mean square of the row or column in the data matrix. The ICC metric ranges from 0 (indicating high variability and low consistency amongst trials) to 1 (indicating low variability and high consistency amongst trials).

### Statistical Analysis

Other statistical analyses were conducted using SPSS 26. Between-group analyses were conducted to determine the extent of differences in intra-individual variability in the retinal response to identical light flashes between the schizophrenia and healthy control groups. We conducted an independent samples *t*-test in order to compare mean ICC values between groups during the photopic condition. To determine if there were between-group differences in median ICC within the scotopic condition, a Mann–Whitney *U*-test was conducted, as the ICC values among the schizophrenia group were not normally distributed. As an additional measure of trial-by-trial variability, we examined variability in peak activity across trials in the a- and b-wave time windows. These analyses did not use the typical a- and b-wave amplitude metrics, which are difference scores, but rather, the minimum and maximum values, respectively, within the a- and b-wave timeframes, which are the primary determinants of a- and b-wave peak values, and included all datapoints (i.e., outliers that were excluded from between-group ICC analyses). This was thus a conservative approach, but one that guaranteed that all variability was captured. Mann–Whitney *U*-tests (used due to violations of normality in the SD and CV distributions) were used to compare the two groups on these values. To examine the relationship between intra-individual variability and strength of the retinal response, correlations were conducted between individual ICC scores and ERG parameters (a- and b-wave metrics) and the Fisher's *r* to *z* transformation was used to investigate the presence of group differences in these relationships. Lastly, we conducted correlations to examine the relationships between ICC and PANSS symptom severity scores.

## Results

### Demographic Information

All demographic information is presented in Table 1. The two groups did not differ on any demographic variables, including gender composition, age, race and ethnicity composition, and education.

### ICC and ERG Amplitude

**Figure 1:**
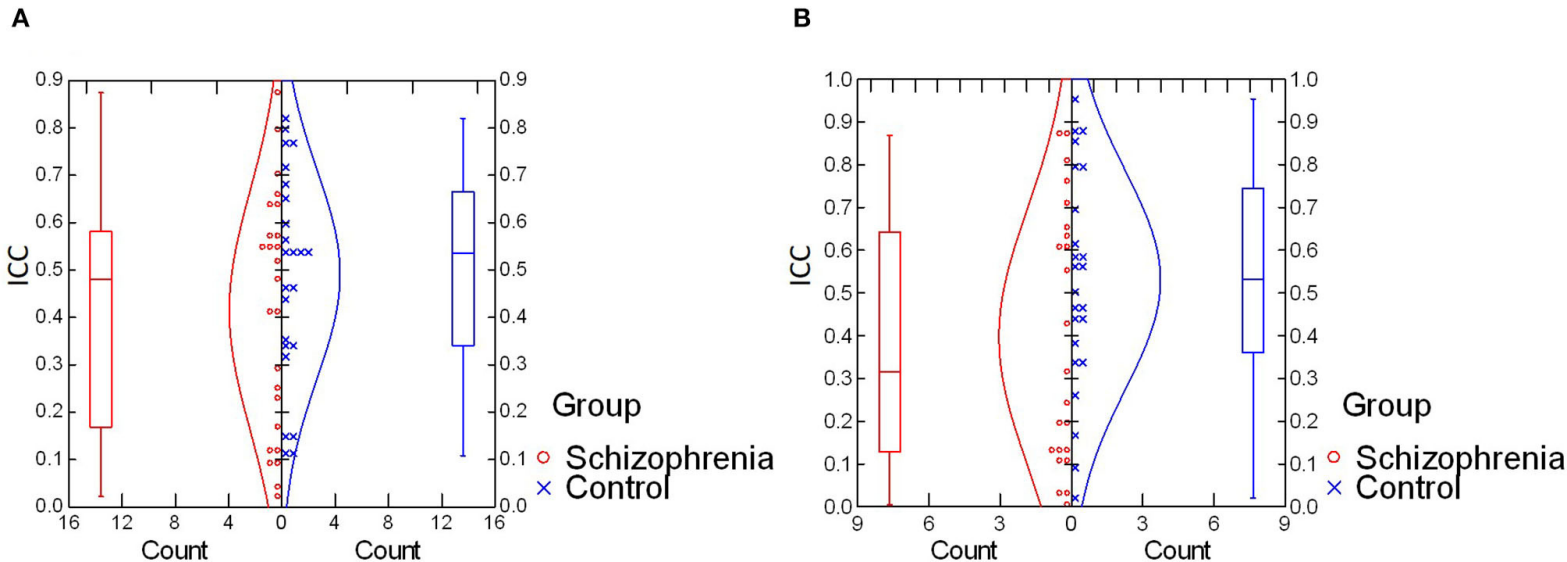
**(A)** Comparison of intra-individual variability (ICC values) in photopic condition. No significant difference was found for mean ICC values between schizophrenia and control groups [*t*_(47)_ = −1.12, *p* = 0.27; *d* = 0.34]. **(B)** Comparison of intra-individual variability (ICC values) in the scotopic condition. No significant difference was found in median ICC values between schizophrenia and control groups (*U* = 208, *z* = 1.45, *p* = 0.15; η^2^ = 0.03). ICC, intraclass correlation coefficient.

For intra-individual variability in the retinal response during the photopic condition, an independent samples *t*-test revealed no significant difference in mean ICC values between schizophrenia (*M* = 0.41, *SD* = 0.25) and control (*M* = 0.49, *SD* = 0.22) groups, *t*_(47)_ = −1.12, *p* = 0.27; *d* = 0.34. In the scotopic condition, a Mann–Whitney *U*-test also demonstrated no significant between-group differences in median ICC values for schizophrenia (*Mdn* = 0.32) and control (*Mdn* = 0.53) groups, *U* = 208, *z* = 1.45, *p* = 0.15; η^2^ = 0.03. These results are illustrated in Figure 1.

We observed no between-group differences in photopic a-wave SD (*p* = 0.47), photopic a-wave CV (*p* = 0.26), photopic b-wave SD (*p* = 0.30), scotopic a-wave SD (*p* = 0.36), scotopic a-wave CV (*p* = 0.42), scotopic b-wave SD (*p* = 0.93), or scotopic b-wave CV (*p* = 0.21). Median values of these variables are listed in Table 2. Although results indicated that the schizophrenia group photopic b-wave CV (*Mdn* = 0.79) was greater, at a marginally significant level, than that of the control group (*Mdn* = 0.58), *U* = 191, *z* = −2.18, *p* = 0.03, all other tests indicated the absence of group-differences in trial-by-trial variability.

**Table 2:** Descriptive statistics for study metrics.

**Variable**	**SZ**		**CON**	
	***M (SD)***	***Mdn***	***M (SD)***	***Mdn***
**Photopic condition**			
ICC	0.42 (0.25)	0.48	0.49 (0.22)	0.53
Amplitude a-wave	−23.58 (8.29)	−22.4	−30.42 (7.43)	−31.80
Trial-by-trial a-wave *SD*	25.83 (18.06)	21.00	23.53 (18.19)	15.61
Trial-by-trial a-wave *CV*	−0.59 (0.36)	−0.53	−0.50 (−0.30)	−0.40
Amplitude b-wave	32.26 (11.07)	31.70	42.90 (12.39)	41.35
Trial-by-trial b-wave *SD*	23.67 (24.72)	13.46	17.32 (15.79)	10.62
Trial-by-trial b-wave *CV*	1.00 (0.63)	0.79	0.66 (0.34)	0.58
**Scotopic condition**			
ICC	0.40 (0.30)	0.32	0.53 (0.26)	0.53
Amplitude a-wave	−10.30 (8.26)	−8.12	−7.66 (6.06)	−7.60
Trial-by-trial a-wave *SD*	22.02 (23.34)	16.67	19.60 (29.63)	10.43
Trial-by-trial a-wave *CV*	−0.69 (0.29)	−0.63	−0.65 (0.39)	−0.62
Amplitude b-wave	41.87 (17.76)	43.65	52.25 (13.25)	52.05
Trial-by-trial b-wave *SD*	25.77 (19.87)	17.73	27.60 (25.43)	18.46
Trial-by-trial b-wave *CV*	0.51 (0.29)	0.53	0.42 (0.26)	0.40

*SZ, schizophrenia; CON, control; ICC, intraclass correlation coefficient*.

**Figure 2:**
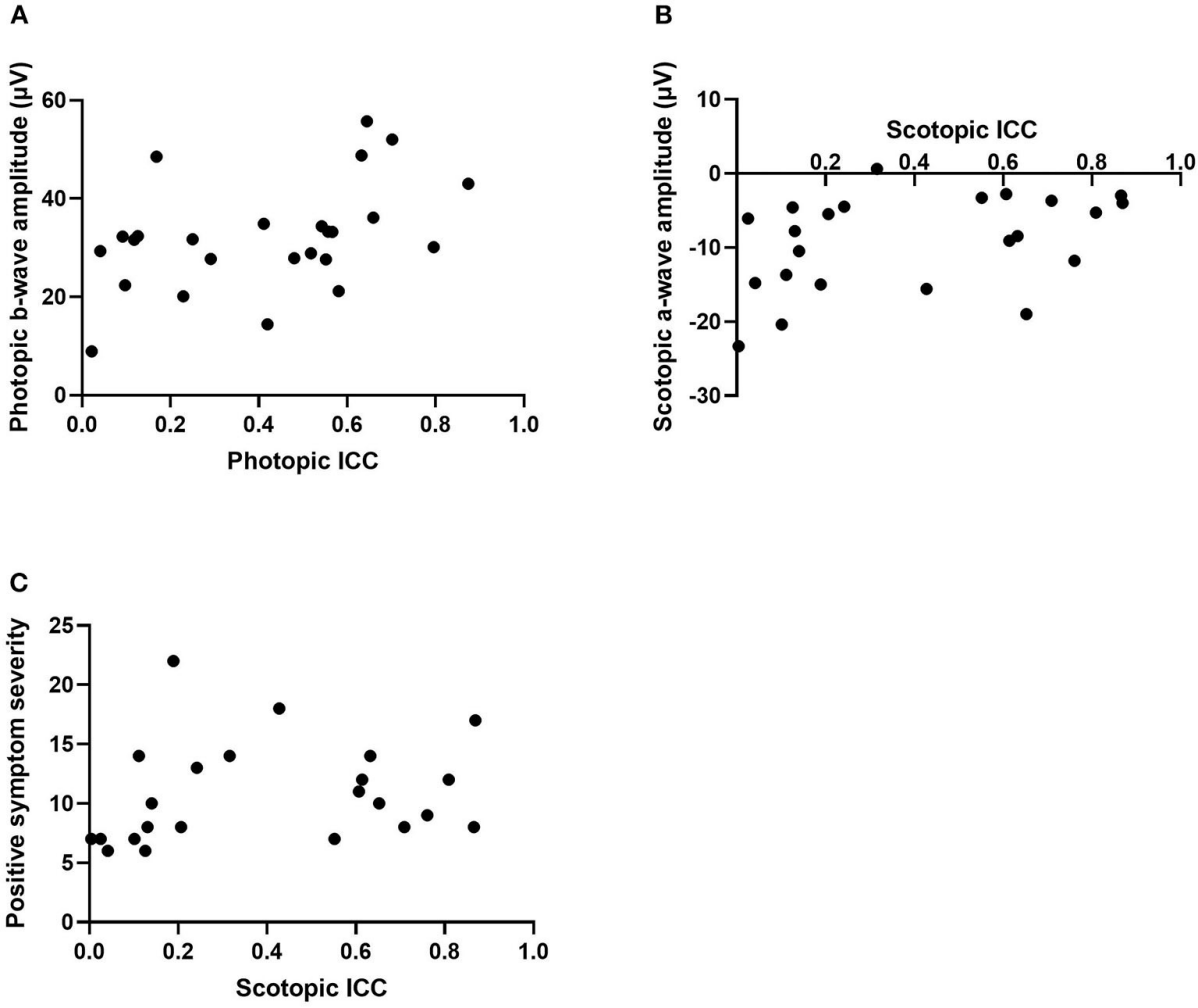
Scatterplots representing the relationship between intra-individual variability (ICC) and photopic b-wave amplitude in microvolts **(A)**, scotopic a-wave amplitude in microvolts **(B)**, and positive symptom severity within the scotopic condition **(C)**. ICC, intraclass correlation coefficient.

Correlational analyses were conducted to examine the relationships between ICC and ERG values. For the schizophrenia group in the photopic condition, a-wave amplitude was negatively correlated with ICC at a trend level of significance [*r*_(23)_ = −0.38, *p* = 0.06] and b-wave amplitude was significantly correlated with ICC [*r*_(23)_ = 0.44, *p* = 0.03; see Figure 2A]. That is, larger a- and b-wave amplitudes were associated with less trial-to-trial variability in the waveform trajectory. However, in the scotopic condition, a-wave amplitude was positively correlated with ICC among schizophrenia patients [*r*_*s*__(21)_ = 0.45, *p* = 0.03; see Figure 2B], indicating that larger a-wave amplitudes were associated with greater variability. ICC was not significantly correlated with b-wave amplitude in the scotopic condition within the schizophrenia group (*p* = 0.70). There were no significant correlations between ICC and ERG latency measures for the schizophrenia group in either the photopic or scotopic conditions (*p*'s > 0.30). For the healthy control group, there were no significant correlations between ICC and any ERG indices, with all *p* > 0.19. We also found no differences between schizophrenia and healthy control groups in the strength of the relationships between intra-individual variability (ICC) and magnitude of the retinal response (a- and b-wave amplitude) for both photopic and scotopic conditions (*p*'s > 0.17).

### Correlations With Symptoms

Correlations were also conducted to determine associations between ICCs in the patient group and PANSS symptom severity scores. Within the photopic condition, positive and disorganized symptom severity were positively associated with ICC within the schizophrenia group at a trend level of significance (*r* = 0.37, *p* = 0.07; *r* = 0.38, *p* = 0.06). This indicates that greater symptom severity was associated with less variability. In the scotopic condition, positive symptoms were positively correlated with ICC (*r*_*s*_ = 0.42, *p* = 0.04; see Figure 2C). That is, greater positive symptom severity was significantly associated with less variability. There were no significant correlations found between ICC, in both photopic and scotopic conditions, and the negative (*p*'s > 0.37), excitement (*p*'s > 0.11), and depressed (p's > 0.87) symptom severity cluster scores.

## Discussion

The primary aim of this study was to compare people with schizophrenia and healthy controls on their level of intra-individual variability within the retinal response to light stimuli. We examined data from a photopic condition in which between-group differences in the magnitude of ERG amplitudes were previously found between schizophrenia and healthy control groups, as well as from a scotopic condition, in which between-groups differences in ERG amplitude were not observed. Contrary to our expectations, we did not find a significant difference in intra-individual variability in retinal activity between groups in either condition tested. In particular, we found no differences in trial-by-trial variability across the entire temporal sequence of the retinal response, as measured by the ICC, as well as no differences in trial-by-trial variability of peak values within a- and b-wave time windows, as measured by the SD and CV, for nearly all analyses. Although we observed a greater CV for photopic b-wave amplitude, this finding was marginally significant and would not survive even the most liberal test for multiple comparisons. Thus, results were generally consistent in demonstrating the absence of increased intra-individual variability in retinal activity in individuals with schizophrenia when compared to a healthy control group.

Although previous studies indicated greater variability in behavioral and electrophysiological responses during cognitive tasks in individuals with schizophrenia when compared to healthy controls (1, 2, 6, 7, 9), no prior study had examined intra-individual variability in the retinal response in schizophrenia. Our data suggest that greater inconsistency in response to repetitive identical stimuli is not present at the sensory level of visual processing in people with schizophrenia.

We also examined the relationship between intra-individual variability and ERG amplitude and latency measures. Within the schizophrenia group, findings indicated that larger b-wave peak amplitudes were associated with less trial-by-trial variability within the photopic condition. A similar result, but at a trend level was found for the relationship between more pronounced a-wave amplitude and less variability in the photopic condition. Interestingly, these correlations were not observed in the control group, which suggests that previous findings indicating reduced a- and b-wave activity in schizophrenia [see (13) for a review] may reflect a subgroup of individuals with schizophrenia with high retinal response variability. This is further supported by the absence of group-level differences in the strength of the relationship between intra-individual variability and retinal response between individuals with schizophrenia and healthy controls. However, in the scotopic condition, attenuated a-wave amplitudes were associated with less variability among the schizophrenia group, which was the opposite of what was predicted. Therefore, caution is indicated in reaching conclusions about the relationship, if any, between a- and b-wave amplitudes and intra-individual variability in people with schizophrenia at this time.

Additional analyses assessing the relationship between symptom severity and retinal variability also revealed unexpected findings. We found that people with greater positive symptom severity displayed less variability in their ERG response within the scotopic condition. We also found results of marginal significance in the photopic condition whereby people with greater positive and disorganized symptom severity demonstrated less variability in their retinal response. Again, these findings contradict the predicted outcome, and there is not a firm basis in the literature from which to interpret them. Given that the statistically significant findings were uncorrected for multiple comparisons, additional research is warranted to understand the relationship between symptom severity and trial-by-trial variability.

This study involved several limitations which should be addressed in future studies. First, the light conditions that were tested only focused on variability in ERG activity reflecting photoreceptor and bipolar-Müller cell activity. Additional studies should also focus on variability in the photopic negative response of the fERG within schizophrenia, which is a component that reflects retinal ganglion cell activity (31) and has been shown to be reduced within the disorder (18). Second, this study included relatively small sample sizes that did not allow for analyses comparing subgroups of participants with schizophrenia with considerably reduced ERG values to participants with ERG data closer to normative values. Third, the scotopic condition tested only included five trials, compared to 30 trials in the photopic condition. While greater temporal spacing between trials is needed in scotopic compared to photopic conditions in order to re-establish dark adaptation after a light flash, the relatively small number of trials in the photopic condition may have limited the ability to reliably detect individual differences in intra-individual variability. Therefore, future studies examining intra-individual variability in scotopic conditions should examine variability across a greater number of trials if possible.

Taken together, results demonstrated no difference in trial-by-trial variability in the retinal response to identical light flashes when comparing people with schizophrenia and healthy controls. This finding was consistent for a light-adapted condition, in which people with schizophrenia showed reduced ERG amplitude when compared to controls, as well as a dark-adapted condition, in which no between-group differences in ERG were found. One implication of these data is that excessive noise in the visual system in people with schizophrenia may arise at a post-retinal (or, post bipolar cell level). If this finding is replicated in a larger sample and with a wider range of ERG testing conditions, it should motivate additional studies to explore at what point in the visual system excessive noise due to intra-individual variability in neural responses is observed.

## Data Availability Statement

The dataset generated for this study can be found in the Open Science Framework repository at: https://osf.io/tn3xa/ Open Science Framework.

## Ethics Statement

The study involved human participants and was reviewed and approved by Rutgers University Health Sciences Institutional Review Board. The patients/participants provided their written informed consent to participate in this study.

## Author Contributions

SF co-developed the concept of the project, conducted the statistical analyses, and wrote the majority of the manuscript. ME wrote the Matlab software for the trial rejection routines, wrote portions of the Methods section, and assisted in editing the manuscript. DD collected all original data for the study, wrote portions of the Methods section, and edited portions of the manuscript. SS co-developed the concept of the project, wrote the software to calculate ICCs based on trial-by-trial data, and wrote and edited portions of the manuscript. All authors contributed to the article and approved the submitted version.

## Conflict of Interest

The authors declare that the research was conducted in the absence of any commercial or financial relationships that could be construed as a potential conflict of interest.
